# Impact of Edible Insect Polysaccharides on Mouse Gut Microbiota: A Study on White-Spotted Flower Chafer Larva (*Protaetia brevitarsis seulensis*) and Silkworm Pupa (*Bombyx mori*)

**DOI:** 10.3390/foods14010006

**Published:** 2024-12-24

**Authors:** Joon-Ha Lee, Hyojung Son, Sathiyamoorthy Subramaniyam, Hyun-Jung Lim, Sohyun Park, Ra-Yeong Choi, In-Woo Kim, Minchul Seo, Hae-Yong Kweon, Yongsoon Kim, Seong-Wan Kim, Jong-Soon Choi, Younhee Shin

**Affiliations:** 1 Department of Agricultural Biology, National Institute of Agricultural Sciences, Rural Development Administration, Wanju 55365, Republic of Koreatarupa@korea.kr (S.-W.K.); 2 Research and Development Center, Insilicogen Inc., Yongin 16954, Republic of Korea; 3 Department of Family Medicine, College of Medicine, Kosin University, Busan 49267, Republic of Korea

**Keywords:** entomophagy, food safety, gut microbiome, insect polysaccharide, prebiotics, probiotics, PCR, Lactococcus, *Protaetia brevitarsis seulensis*, *Bombyx mori*

## Abstract

The increasing global population and the environmental consequences of meat consumption have led to the exploration of alternative sources of protein. Edible insects have gained attention as a sustainable and nutritionally rich meat alternative. We investigated the effects of two commonly consumed insects, *Protaetia brevitarsis seulensis* larva and *Bombyx mori* pupa, on beneficial gut microbiota growth, using whole 16s metagenome sequencing to assess diet-associated changes. Seven-week-old female C57BL/6J mice were administered the edible insects, along with fracto-oligosaccharide (FOS) as a positive control and sham (phosphate buffer saline (PBS)) as a negative control, to assess the relative abundance of insect-diet-associated gut microbes. In total, 567 genera and 470 species were observed, and among these, 15 bacterial genera were differentially abundant in all three groups. These results show that among the two insects, *Bombyx mori* pupa polysaccharides have a greater ability to regulate beneficial probiotics and next-generation probiotics. In particular, *Lactococcus garvieae*, which has promising effects on the gastrointestinal tracts of humans and animals, was significantly enriched in both *Protaetia brevitarsis seulensis* larva and *Bombyx mori* pupa polysaccharides, similar to fracto-oligosaccharide. The results suggest that the consumption of these insects, particularly polysaccharides, can enhance the growth of beneficial gut microbes, potentially leading to improved overall health in healthy populations.

## 1. Introduction

According to the United Nations, the global population is projected to rise to approximately 9.8 billion by 2050, presenting significant challenges for food security and environmental sustainability [1]. To meet the increasing demand for meat, which is associated with increased greenhouse gas emissions and other environmental concerns, alternative protein sources are being sought [2]. The current average per capita meat consumption is forty-three kilograms, emphasizing the importance of protein sources in diets [1]. As a result, edible insects have gained attention as a sustainable and nutritionally rich option with potential benefits for both human and animal health [3]. Insect farming/rearing has become popular for food and feed production because it is more cost-effective than other animal and plant protein sources [4]. Additionally, the incorporation of insects into food varies widely based on indigenous cultural practices [5]. Globally, there are approximately 2205 edible insects, of which 42% are found in Asia [5]. Insects contain numerous therapeutic molecules beneficial for humans; these molecules are influenced by the insects’ unique living habits and environment. Furthermore, the use of insects in food production is increasing because they are rich in proteins, healthy fats, vitamins, minerals, and other essential nutrients easily absorbed and utilized by the human body [6,7]. The most commonly consumed insect proteins are crickets, mealworms, black soldier fly larvae, silkworm pupae, grasshoppers, and locusts, with regional preferences and traditional practices influencing consumption patterns [8,9,10]. These proteins are used in various food products, including snacks, pasta, baked goods, shakes, protein bars, and alternative meat products [4]. As insect-based foods gain consumer acceptance and the demand for insect-based products rises, it will be essential to focus on entomophagy resource production and food safety screening [3,11,12].

The interplay between prebiotics, probiotics, and postbiotics is crucial for maintaining healthy gut microbiota, which is vital for overall metabolic health. This interplay regulates the immune system, protects the integrity of the gut barrier, and prevents dysbiosis, ultimately having positive effects on host health [13,14]. In particular, prebiotics are known for their role in promoting the growth and activity of beneficial gut bacteria. As non-digestible food components, they act as substrates that are selectively utilized by host microorganisms, conferring health benefits [15]. Studies have shown that various substances, such as dietary fibers and specific insect extracts/protein powders, can function as prebiotics by stimulating the proliferation of beneficial bacteria, including Lactobacillus and Bacteroides species, in the gastrointestinal tract [16,17]. However, prebiotics generally support beneficial bacteria without always directly correlating with increased growth or biofilm formation, as observed in a study of oral bacteria change [18]. In addition to supporting human health, prebiotics influence the gut microbiota of poultry, improving growth performance and immune system development [19]. Overall, prebiotics enrich beneficial gut bacteria (i.e., probiotics), contributing to a balanced intestinal microbiota and overall host health. More specifically, the edible insects are an excellent source of prebiotics, which promote beneficial probiotic bacteria growth to produce various postbiotics and bioactive compounds, such as short-chain fatty acids, which are essential for gut health [20,21,22]. Specifically, chitin, a major polysaccharide fiber abundant in edible insects, has several health-promoting properties, including antioxidant, antidiabetic, antiobesity, and anticancer activities [20].

Additionally, the advent of next-generation sequencing has revolutionized the technical aspects of screening, providing researchers with a powerful tool for delving into the nuanced connections between dietary intake and the microbial communities residing in the gut. This technology offers cost-effective insights into the factors’ complex interactions under various conditions [23,24]. Furthermore, it enables researchers to examine multifaceted connections between the gut microbiota and other organs, such as the brain, liver, and immune system, enhancing our understanding of the gut–organ axis [25,26]. Notably, there is growing interest in the gut microbiota’s effects on immunological processes while consuming different insect-based diets [27,28,29]. Prior to the advent of high-throughput next-generation sequencing, scientists relied on targeted approaches to investigate this system [30,31]. These earlier methods were limited in their ability to provide a holistic understanding of this system, resulting in slower progress compared to current techniques [31,32]. The human gut harbors a diverse array of microorganisms that form a complex, dynamic ecosystem influenced by external factors like diet and environment [31]. To fully comprehend the entire gut association system, it is crucial to analyze data from the complete gastrointestinal tract rather than focusing on individual components [24,30]. This comprehensive approach is particularly effective when combined with advanced statistical and computational modeling outcomes [33].

Drawing on existing knowledge, this study will explore the implications of integrating two edible insect species into food production diets: *Protaetia brevitarsis seulensis* larva (Pbs larva), also referred to as white-spotted flower chafer larva, and *Bombyx mori* pupa (Bm pupa), commonly known as Silkworm Pupa, which is more commonly used in sericulture industries for silk production and has recently gained attention as a food supplement [34]. These insects have traditionally been used in Chinese and Korean medicine; Pbs larvae, also referred to as Gumbengi, have been utilized for addressing digestive disorders, inflammatory conditions, and skin problems. Bm pupa have been applied to treat conditions such as arthritis, joint pain, skin diseases, and gastrointestinal issues. Moreover, past research has shown that both insect powders demonstrate immunomodulatory effects in mouse models [35,36,37]. These insects serve not only as food sources but also as raw materials for pharmaceutical products, with past studies investigating their therapeutic benefits. Pbs larva powder has been evaluated both as a potential food supplement resource [38,39] and for its therapeutic effects [40], including benefits for treating osteoarthritis [41,42,43], platelet aggregation [44,45], radiation therapy protection [46,47], neuroprotection [48], and liver protection [49], as well as applications in biomaterials such as chitosan [40] and other food packaging and preservation materials [50,51]. Similarly, Bm pupa powder has been evaluated for its prebiotic effects [52], menopause relief [53], hypertension management [54], its antiobesity properties [55], atopic dermatitis treatment [56], liver protection [57,58], immune response enhancement [59,60], and applications in biomaterials [61] and food packaging [62]. However, both insect powders have been assessed for various applications and therapeutic uses, but they have not been investigated for their prebiotic effects, which influence the growth of beneficial microbiota through changes in gut microbiota. The previously assessed factors were assessed with fracto-oligosaccharide (FOS), a well-established prebiotic that supports gut health by promoting the growth of beneficial bacteria and inhibiting harmful pathogens [63,64,65], as a positive control. Additionally, the nutritional profiles of edible insects were evaluated based on their raw-weight fiber contents, with Lepidoptera and Coleoptera taxonomical-order insects being widely consumed according to edible insect catalogs [20]. In our study, we employed polysaccharides (PSs) extracted from Pbs larva in the order Coleoptera and Bm pupa in the order Lepidoptera to evaluate their prebiotic effects on the abundance of probiotic microbes in mouse models. Furthermore, this research also seeks to expand the understanding of prebiotic sources beyond traditional plant-derived fructo-oligosaccharides by investigating these novel insect-derived polysaccharides. The long-term objective is to establish the feasibility of using these insect-derived polysaccharides as novel prebiotic ingredients in functional foods or dietary supplements.

## 2. Materials and Methods

### 2.1. Insect Samples

*Protaetia brevitarsis seulensis* (Kolbe, 1886) and *Bombyx mori* (Backokjam) were reared at the insect-rearing facilities of the National Institute of Agricultural Sciences in Wanju, Republic of Korea. The white-spotted flower chafer larvae were reared in a constant rearing room at 25 ± 1 °C, 65 ± 5% relative humidity (RH), and a 16 h light/8 h dark photoperiod cycle on fermented oak sawdust. The last instar larvae were harvested, washed, boiled, and hot air-dried. The hot air-dried larvae were then pulverized using a mortar and pestle to create a powder (<1 mm), which was used for aqueous extraction and PS purification. The silkworms were reared under standard conditions of temperature (24–27 °C) and humidity (70–90%) and a 16 h light/8 h dark photoperiod cycle. The silkworm pupae were collected from the cocoons after dissection and stored at −70 °C until use. The frozen pupae were pulverized using a mortar and pestle with liquid nitrogen, and the powder was used for aqueous extraction and PS purification. The weight of a dried white-spotted flower chafer larva is ~0.5 g. An average of 20 larvae were used to obtain 10 g. Similarly, the weight of each silkworm pupa was about 1.9 g. An average of five pupae were used to obtain 10 g of powder.

### 2.2. Chemicals and Reagents

Chloroform (99.5%, DAEJUNG CHEMICALS & METALS Co., LTD, Siheung, Republic of Korea), n-Butanol (99%, DAEJUNG CHEMICALS & METALS Co., LTD, Siheung, Republic of Korea), ethyl alcohol (99.45%, Sigma-Aldrich, St. Luis, MO, USA), phosphate-buffered saline (PBS; Caisson Labs, Inc. Smithfield, UT, USA), 3-Methyl-1-phenyl-2-pyrazoline-5-one (99%, Sigma-Aldrich, St. Luis, MO, USA), Trifluoroacetic acid (99%, Sigma-Aldrich, St. Luis, MO. USA), sodium hydroxide (93%, DUKSAN GENERAL SCIENCE Co., LTD, Seoul, Republic of Korea), hydrochloric acid (37%, DUKSAN GENERAL SCIENCE Co., LTD, Seoul, Republic of Korea), acetonitrile (DUKSAN GENERAL SCIENCE Co., LTD, Seoul, Republic of Korea), and ammonium acetate (97%, Sigma-Aldrich, St. Luis, MO, USA) were the chemical reagents used.

### 2.3. Preparation of Insect Polysaccharides

Insect powder, weighing 10 g, was combined with 100 mL of water. The mixture was subjected to a shaking water bath at 70 °C for 6 h and then centrifuged at 3000 times gravity for 10 min. The supernatant was filtered through a 0.45-micrometer syringe filter and underwent treatment with Sevag solution, which consisted of chloroform and n-butanol in a 4:1 ratio, before centrifugation to remove protein and organic residues. The Sevag solution was prepared in the fume hood, and the solution was disposed in the waste liquid tank after treatment. The resulting supernatant, which contained polysaccharides, was mixed with 95% ethanol in a shaking incubator at 4 °C for 12 h. The precipitated polysaccharides were then collected through centrifugation and lyophilization. Water-soluble polysaccharides were isolated from the aqueous extract of Pbs larvae and Bm pupae. The polysaccharides were stored at 4 °C until use.

### 2.4. Animal Model

The study protocol was approved by the Institutional Animal Care and Use Committee (IACUC) of the National Institute of Agricultural Sciences (NAS-202302). Seven-week-old female C57BL/6J mice, weighing 20–25 g, were procured from Central Lab. Animal Inc. (Seoul, Republic of Korea) and acclimated to a temperature of 22 ± 2 °C, a humidity of 40 ± 5%, and 12 h light–dark cycle conditions for one week. They were then fed a standard diet (Purina, Co., LTD, Seongnam, Republic of Korea) that contained 20% crude protein, 4.5% crude fat, 6% crude fiber, 7% crude ash, 0.5% calcium, and 1% phosphorus and sterilized water ad libitum for a week as an adaptation period. The mice were subsequently divided into four groups (*n* = 8) as follows: control (phosphate buffer saline (PBS), FOS (50 mg/kg), Pbs larva PS (50 mg/kg), and Bm pupa PS (50 mg/kg); eight mice were included in each cage, and the groups were housed in separate cages. The mice were administered FOS, Pbs larva PS, and Bm pupa PS intragastrically at doses of 50 mg/kg/day, respectively. The control and treatment groups were treated with the same volume of PBS. The control ingested 100 microliters of PBS via oral administration. All mice received a standard diet (Purina, Co., LTD, Seongnam, Republic of Korea) and tap water ad libitum for 6 weeks. Body weight was measured every 2 days during the feeding period. For six weeks, the mice were given free access to food and water. The mice were sacrificed at the end time point (six weeks, *n* = 8 each) via CO_2_ asphyxiation. The average weights of the mice at the sacrifice times were 28.20 ± 0.48 g (*p* > 0.05). After dissection, the colons were collected for microbial analysis. Thirty-two colon samples were used for the gut microbiota analysis, and each sample was chosen based on the average weight on the sacrifice week. All colon samples were prepared for NGS analysis. The tissue and stool samples were immediately frozen at −80 °C in a deep freezer after collection.

### 2.5. 16s Library Construction and Sequencing

The DNA from stored samples was extracted using a DNeasyPowerSoil Kit (Qiagen, Hilden, Germany) according to the manufacturer’s instructions. The extracted DNA was quantified using Quant-IT PicoGreen (Invitrogen, Waltham, MA, USA). Further, the 16S metagenomic sequencing library was amplified using the Illumina protocol, starting with an input of 5 ng gDNA. The PCR reaction was performed using 5X buffer, 1 mM of dNTP mix, 500 nM of each of the universal F/R PCR primers, and Herculase II fusion DNA polymerase (Agilent Technologies, Santa Clara, CA, USA). The PCR cycling conditions included a heat activation step of 3 min at 95 °C, followed by 25 cycles of 30 s at 95 °C, 30 s at 55 °C, and 30 s at 72 °C, as well as a final extension step of 5 min at 72 °C. The universal primer pair with Illumina adapter overhang sequences used for the first amplification was as follows: 16S Amplicon PCR Forward Primer 5′ TCGTCGGCAGCGTCAGATGTGTATAAGAGACAGCCTACGGGNGGCWGCAG and 16S Amplicon PCR Reverse Primer 5′ GTCTCGTGGGCTCGGAGATGTGTATAAGAGACAGGACTACHVGGGTATCTAATCC. After the first PCR, the product was purified with AMPure beads (Agencourt Bioscience, Beverly, MA, USA) and then PCR-amplified for final library construction containing the index using the NexteraXT Indexed Primer. The cycle conditions for the second PCR were the same as those for the first PCR, except for 10 cycles. The PCR products were purified using AMPure beads, and the final purified product was quantified using qPCR according to the qPCR Quantification Protocol Guide (KAPA Library Quantification kits for Illumina Sequencing platforms) and qualified using TapeStation D1000 ScreenTape (Agilent Technologies, Waldbronn, Germany). Finally, the library was sequenced using MiSeq™ (Illumina, San Diego, CA, USA).

### 2.6. Metagenome Sequence Process and Differential Abundance Analysis

The 16S V3-4 fragments from each library were analyzed to determine their microbial communities and differential microbial abundances using the QIIME2 2024.02 package [66]. These steps included using preprocessing to remove non-informative sequences and merging and correcting the paired sequences. The merged sequences were then clustered into operational taxonomic units (OTUs) using the DADA2 method [67] and annotated with the GSR database (Greengenes, SILVA, and RDP databases) to obtain detailed taxonomic information for each OTU [68]. Alpha- and beta-diversity analyses were performed to understand sample diversity, and the relative and differential abundances were analyzed using the Analysis of Compositions of Microbiomes with Bias Correction (ANCOM-BC) statistical method, analyzing each pair of conditions individually [33].

### 2.7. Microbiota Disease Association Analysis

The ANCOM-BC assessment identified differentially present microorganisms, which were subsequently mapped to the Gut Microbial Metabolite Association with Disease (GMMAD) [69] and gutMDisorder v2.0 databases [70]. These databases provide highly specialized insights on human gut microbiota and associated diseases, drawing on the literature and publicly available microbiota sequencing repositories.

### 2.8. Determination of Monosaccharide Compositions

**Figure 1:**
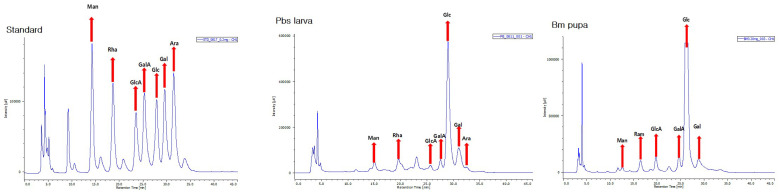
HPLC profile of the monosaccharide composition of the polysaccharides following hydrolysis and derivatization. Standard monosaccharides, Pbs larva, and Bm pupa monosaccharides. Mannose (Man), rhamnose (Rha), glucuronic acid (GlcA), galacturonic acid (GalA), glucose (Glc), galactose (Gal), and arabinose (Ara).

The isolated polysaccharides (0.02 g) underwent hydrolysis with 2 mL of trifluoracetic acid and were autoclaved at 121 °C. The resulting mixture was neutralized using 2 mL NaOH before being centrifuged at 6000 rpm for 10 min. From this mixture, 0.5 mL of supernatant was extracted and combined with 0.5 mL of 1-phenyl-3-methyl-5-pyrazolone (PMP) in 0.5 mL of MeOH and 0.3 mL of NaOH. This solution was derivatized for one hour at 70 °C, subsequently cooled, and neutralized with 0.3 mL of HCl. To eliminate PMP, 5 mL of chloroform was added, and the mixture was vortexed. The prepared samples were then analyzed using HPLC (UV-2075 plus, Jasco International Co., Ltd., Tokyo, Japan). The polysaccharide content was measured by injecting the samples into an Athena C18 reverse-phase column (250 mm × 4.6 mm, 5 μm, CNW Technologies, Dusseldorf, Germany) and using a UV detector set at a 245 nm wavelength. The gradient elution consisted of solvent A: 8% acetonitrile in 0.1 M ammonium acetate buffer (pH 5.5) and solvent B: 30% acetonitrile in 0.1 M ammonium acetate buffer (pH 5.5), as previously described [71]. The column oven temperature was set at 30 °C, with elution at a flow rate of 0.8 mL/min. The HPLC profiles are given in Figure 1.

## 3. Results

### 3.1. Isolation of Insect Polysaccharides and Monosaccharide Compositions

We obtained the crude polysaccharides from Pbs larva and Bm pupa through deproteinization followed by water extraction and ethanol precipitation. The yields of crude polysaccharides were 16.50 ± 0.56% (Pbs PS) and 10.87 ± 0.60% (Bm PS), respectively. Seven or six monosaccharides were identified in the purified polysaccharide of Pbs larva and Bm pupa via HPLC (Figure 1). Six of these polysaccharides were found to be common among the polysaccharides of Pbs larva and Bm pupa. However, arabinose was detected only in the polysaccharides of Pbs larva. The polysaccharides of Pbs larva and Bm pupa consist of abundant glucose in their monosaccharide composition (Table 1).

### 3.2. Metagenome Sequencing and Taxonomical Profiles

**Figure 2:**
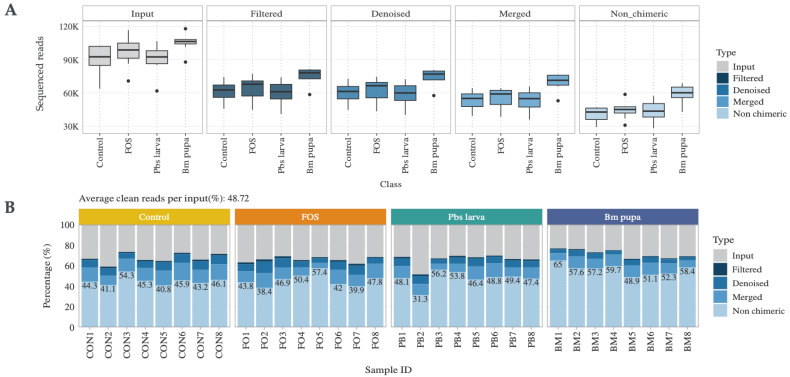
Summary graphs of sequencing data: (**A**) cumulative processed sequence summary; (**B**) sequenced reads from individual samples.

**Figure 3:**
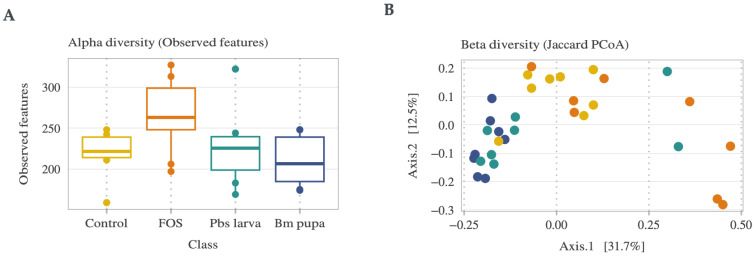
Diversity of sequenced samples: (**A**) alpha diversity calculated from observed features; (**B**) beta diversity calculated from the Jaccard PCoA.

**Figure 4:**
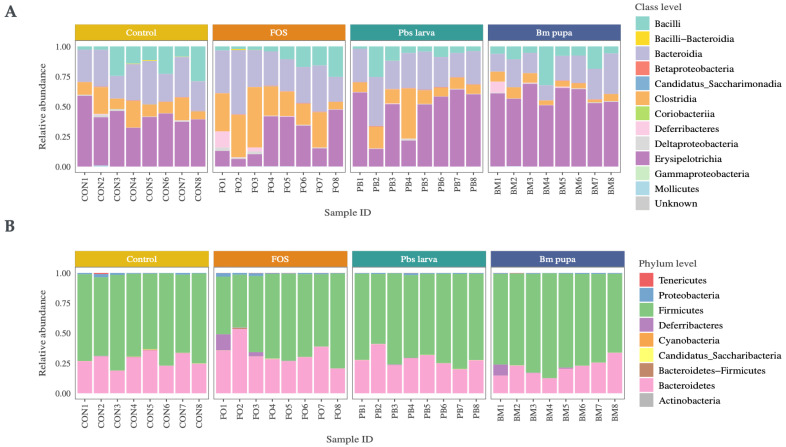
Relative abundances of OTUs at the observed taxonomic level, both at the class level (**A**) and phylum level (**B**).

The process for sequencing the mouse gut microbiota is shown in Figure 2, and the corresponding values are given in Supplementary Table S1. In total, four groups of mice were sequenced using 16s rRNA sequencing to explore the gut microbiota changes that occurred when administering two different insect polysaccharides, i.e., Pbs larva (*Protaetia brevitarsis seulensis* larva) and Bm pupa (*Bombyx mori* pupa), along with FOS (fracto-oligosaccharide) and phosphate-buffered saline (PBS) as the positive and negative controls, respectively. In total, the gut microbiota of 32 mice were sequenced, with each group comprising 8 mice (Figure 2A). A total of 1 GB of sequences was obtained, with an average of 80,000 reads of sequences extracted from all samples. After processing the sequence artifacts until the chimeric reads, as illustrated in Figure 2B, 48.7% of the sequences remained. These processed sequences were subjected to taxonomic annotation, resulting in 969 operational taxonomic units (OTUs) with 567 genera and 470 species. The alpha-diversity, computed using the observed OTUs, revealed that the FOS group contributed to a greater bacterial population than the other groups (Figure 3A). The beta-diversity of the samples, which measured the diversity of observed OTUs with Jaccard PCoA between groups of samples, is illustrated in Figure 3B, demonstrating that the insect polysaccharide-administered samples and the positive control were distinct, while the FOS partially overlapped with the negative control and treatments. Among these, the Erysipelotrichia class of bacteria was abundant in all the samples (Figure 4A). At the phylum level, Bacteroidetes and Firmicutes accounted for approximately 90% of the sequences (Figure 4B).

### 3.3. Relative and Differential Abundances

**Figure 5:**
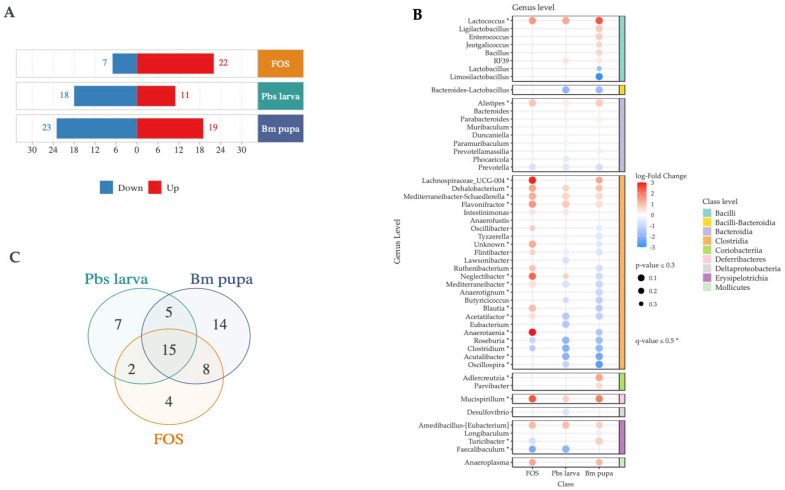
A summary of the relative and differential abundances of mouse gut microbes. (**A**) The number of genera at differential abundances for each group compared with the control is displayed. (**B**) The bubble graph representation of the differential abundance microbes is shown. (**C**) The combination of differential abundance microbes is presented.

**Figure 6:**
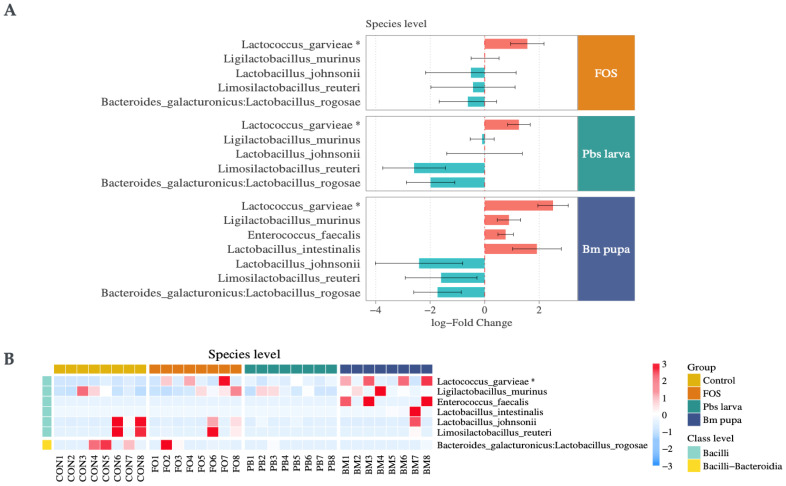
A summary of the species-level differential abundance. (**A**) A list of probiotic species that are differentially abundant. (**B**) The microbial abundance values for the target probiotic species. * significant (q ≤ 0.5).

According to Lin and Peddada [33], it is essential to exercise caution when addressing normalization and differential abundance in metagenomic sequencing. The ANACOM-BC method was employed for differential abundance analysis to better understand the differences between the negative control and the other three groups. In total, we identified 55 bacteria at the genus level that were differentially abundant in all three groups compared with the control (i.e., negative control); thus, the mice were only treated with PBS. This group included OTUs for 65 species, for which the expression values are illustrated in Supplementary Figure S1. The detailed differential abundances of each group in both directions (i.e., up and down) are illustrated in Figure 5A. In total, 29 bacterial genera from FOS, 29 from Pbs larva, and 42 from Bm pupa were significantly differentially abundant (Figure 5A,B). Among those, 15 bacterial genera were differentially abundant in all three groups (Figure 5C). Furthermore, we explored the beneficial microbial, i.e., probiotic differential abundances, for the same species at the species level (Figure 6). Overall, *Lactococcus garvieae* (q-value ≤ 0.5) was significantly enriched in all three diets, i.e., FOS, Pbs larva, and Bm pupa (Figure 5A and Figure 6A,B). *Lactobacillus johnsonii, Limosilactobacillus reuteri*, and *Lactobacillus rogosae* were depleted all three groups compared to the negative control (Figure 5B and Figure 6A,B). *Ligilactobacillus murinus*, *Enterococcus faecalis,* and *Lactobacillus intestinalis* were enriched in the Bm pupa compared to negative control but not in other groups (Figure 6A).

## 4. Discussion

Insect-based diets have shown the potential to act as prebiotics and enhance beneficial gut bacteria [1,11,12], as the chitin and fiber contents of edible insects can serve as substrates for probiotic bacteria in the gut [72,73]. Studies have found that the consumption of insect powders or extracts can stimulate the growth of beneficial bacteria like Lactobacillus and Bifidobacterium species [72,73]. For example, cricket chitosan acts as a potential prebiotic effect assessed under in vitro conditions [73]. In this study, we investigated the aqueous polysaccharides of insects. The monosaccharide composition analysis revealed that the insect polysaccharides consist predominantly of glucose (Glc), with minor quantities of mannose (Man), rhamnose (Rha), glucuronic acid (GlcA), galacturonic acid (GalA), galactose (Gal), and/or arabinose (Ara). These findings suggest that insect polysaccharides contain heteroglycans. Indeed, a recent study reported the characterization of an acidic heteroglycan from Pbs larva, elucidating its structure and immune-enhancing properties [74]. Additionally, an acidic polysaccharide termed “silkrose” from silkmoth pupae has been reported, detailing its molecular weight, monosaccharide composition, and immune-stimulating effect [75]. These results indicate that insect polysaccharides possess dual functionality, exhibiting both prebiotic effects and immune-activating properties. To identify similar activity for the insect polysaccharides, we administered two insect polysaccharides (i.e., *Protaetia brevitarsis seulensis* (Pbs larva) and *Bombyx mori* (Bm pupa)) (Figure 6) to a C57BL/6J mice model. Since C57BL/6J mice, a commonly used strain, are a more stable model, and their widespread use has led to the development of a substantial body of knowledge regarding the mouse gut microbiome, they have become a valuable resource for comparative studies [76,77]. Additionally, to align probiotic promotion with the known reference, we used FOS as a positive control, as explained before. Furthermore, FOS was used to fortify foods with fiber without contributing to any deleterious organoleptic effects, improve the flavor and sweetness of low-calorie foods, and improve the texture of fat-reduced foods. Inulin and oligofructose possess several functional and nutritional properties that may be used to formulate innovative healthy foods for consumers [78]. We used FOS as a reference to assess the beneficial indexes of Pbs larva and Bm pupa insect powder in mice gut microbiota.

We observed similar responses in potential next-generation probiotics such as *Blautia coccoides* [79], *Eubacterium hallii* [80], *Oscillibacter valericigenes* [81], Clostridium spp. [82], and *Lactococcus garvieae* (Figure 6A,B; Supplementary Figure S1). Among these, *Lactococcus garvieae* has promising characteristics and is present in the gastrointestinal tracts of humans and animals [83]. Moreover, we observed the presence of bacteria, such as *Bacteroides acidifaciens*, in the Pbs larva diet-administered group, exhibiting probiotic effects on dextran sodium sulfate (DSS)-induced inflammation, a model commonly used to study inflammatory bowel disease (IBD) [84]. Similarly, *Bacteroides caecimuris*, another bacterium belonging to the same genus, has demonstrated an association with inflammatory mechanisms in the goat gut [85]. Furthermore, *Phocaeicola vulgatus* has been tested for its probiotic potential as a gut microbiota-based therapy for metabolic dysfunction-associated steatosis liver disease [86]. Interestingly, three species from the genus Phocaeicola were also observed after the consumption of Pbs larva polysaccharides (Figure 5A), namely *Phocaeicola massiliensis*, *Phocaeicola sartorii*, and *Phocaeicola vulgatus* (Supplementary Figure S1). Similarly, Bm pupa polysaccharides-administered mice showed positive results regarding the use of widely recognized probiotics, such as *Lactobacillus intestinalis*, *Lactobacillus johnsonii*, *Limosilactobacillus reuteri*, and *Enterococcus faecalis* (Figure 6A,B) [87]. Additionally, other next-generation probiotics, such as Ligilactobacillus, Parabacteroides [88,89], and Adlercreutzia genera [90], also exhibited positive outcomes (Figure 6). For instance, the Parabacteroides genera [88] include species such as *Parabacteroides distasonis*, which is highly associated with diabetes [91] and rheumatoid arthritis [92]; *Parabacteroides goldsteinii*, which is more effective at treating *Helicobacter pylori* [93]; and *Parabacteroides merdae*, which is linked to hypertension and polycystic ovary syndrome [88]. To determine the abundance and disease association patterns of these probiotics and next-generation probiotic microbes in diseases from public databases, we looked for similar patterns with supporting evidence in past studies (Supplementary Figure S2). However, there was difference in the expression; i.e., down and up were different in various studies, as shown in Supplementary Figure S3, as the targeted microbes had equal proportions for enrichment and depletion. Our findings indicated that the consumption of Pbs larva and Bm pupa insect polysaccharides significantly enhanced the growth of beneficial gut microbiota, including species associated with improved digestive and immune functions. A decrease in the abundance of potentially harmful bacteria was also observed. The nutritional contents of these insects, including their rich sources of polysaccharides, may have contributed to these effects. In particular, the more significantly abundant microbe is *Lactococcus garvieae* in the two insect powders, which mimic the FOS known as the fish pathogen; however, it acts as a beneficial microorganism for human and animals [83]. Additionally, it is widely used as a food preservative for industrial purposes [94]. As we know, insect powders (i.e., *Protaetia brevitarsis seulensis* (Pbs larva) [50,51] and *Bombyx mori* (Bm pupa) [62]), which are also used in food packing industries, could enrich *Lactococcus garvieae* to protect the food. Additionally, we found that these insects could be incorporated into functional food products, animal feed, and probiotics, creating potential opportunities for their use in the food industry.

## 5. Conclusions

This study investigated the effects of polysaccharides extracted from two edible insects, *Protaetia brevitarsis seulensis* larva (Pbs larva) and *Bombyx mori* pupa (Bm pupa), on the gut microbiota of mice. The results demonstrated that both insect polysaccharides significantly influenced the composition of the beneficial gut bacteria, with Bm pupa polysaccharides showing a greater ability to regulate beneficial probiotics and next-generation probiotics. Notably, *Lactococcus garvieae*, which has promising effects on the gastrointestinal tracts of humans and animals, was significantly enriched after both Pbs larva and Bm pupa polysaccharide treatment, similar to the positive control fructo-oligosaccharide (FOS). The Pbs larva polysaccharides were associated with increased Bacteroides and Phocaeicola genera abundances, linked to various health benefits. Bm pupa polysaccharides promoted the growth of recognized probiotics such as Lactobacillus and Enterococcus species, as well as next-generation probiotics like Ligilactobacillus and Parabacteroides. These findings suggest that Pbs larva and Bm pupa consumption, particularly consumption of their polysaccharides, can enhance the growth of beneficial gut microbes, potentially improving the overall population health. This study highlights these edible insects’ potential as sustainable and nutritionally rich alternatives to traditional protein sources, providing additional prebiotic benefits. Further research is needed to elucidate the specific mechanisms through which these insect polysaccharides modulate the gut microbiota and explore their potential applications in functional foods, animal feed, and probiotic formulations. Additionally, long-term human studies are needed to confirm the safety and efficacy of these insect-derived polysaccharides for promoting gut health and overall well-being.

## Figures and Tables

**Table 1:** Composition of monosaccharides in the purified polysaccharides. Man, mannose; Rha, rhamnose; GlcA, glucuronic acid; GalA, galacturonic acid; Glc, glucose; Gal, galactose; Ara, arabinose; ND, not detected. All the quantitative values are in (mg/g).

Insects	Man	Rha	GlcA	GalA	Glc	Gal	Ara
Pbs larva	3.80 ± 0.67	7.03 ± 0.25	5.64 ± 0.08	5.34 ± 0.06	50.79 ± 0.22	11.84 ± 0.23	1.47 ± 0.0
Bm pupa	2.10 ± 0.07	7.65 ± 0.15	4.10 ± 0.08	7.82 ± 0.13	472.24 ± 37.04	8.84 ± 0.05	ND

## Data Availability

The complete sequences generated in this study have been deposited in the NCBI Sequence Read Archive under accession no. PRJNA1119635. The original contributions presented in the study are included in the article/Supplementary Materials, further inquiries can be directed to the corresponding author.

## Supplementary Materials

The following supporting information can be downloaded at https://www.mdpi.com/article/10.3390/foods14010006/s1, Figure S1: An illustration depicting differentially abundant microbes with their corresponding relative abundance values, as obtained from QIIME2; Figure S2: The disease associations between the target species and the gut microbiota, as obtained from the Gut Microbial Metabolite Association with Disease (GMMAD) and gutMDisorder v2.0 databases. Figure S3: The expression patterns of target microbe disease associations from the GMMAD and gutMDisorder databases. Table S1: A detailed summary of taxonomic annotations, relative abundances, and differential abundance values.
