# Supplementary material for: Impact of Edible Insect Polysaccharides on Mouse Gut Microbiota: A Study on White-Spotted Flower Chafer Larva (Protaetia brevitarsis seulensis) and Silkworm Pupa (Bombyx mori)
Source: Foods. 2024 Dec 24;14(1):6. doi: 10.3390/foods14010006 (PMC11720208; doi:10.3390/foods14010006)
Supplement: Supplementary file 1 [file foods-14-00006-s001.zip › Suppli_Files/Suppli_Figures.pptx]

## Slide 1
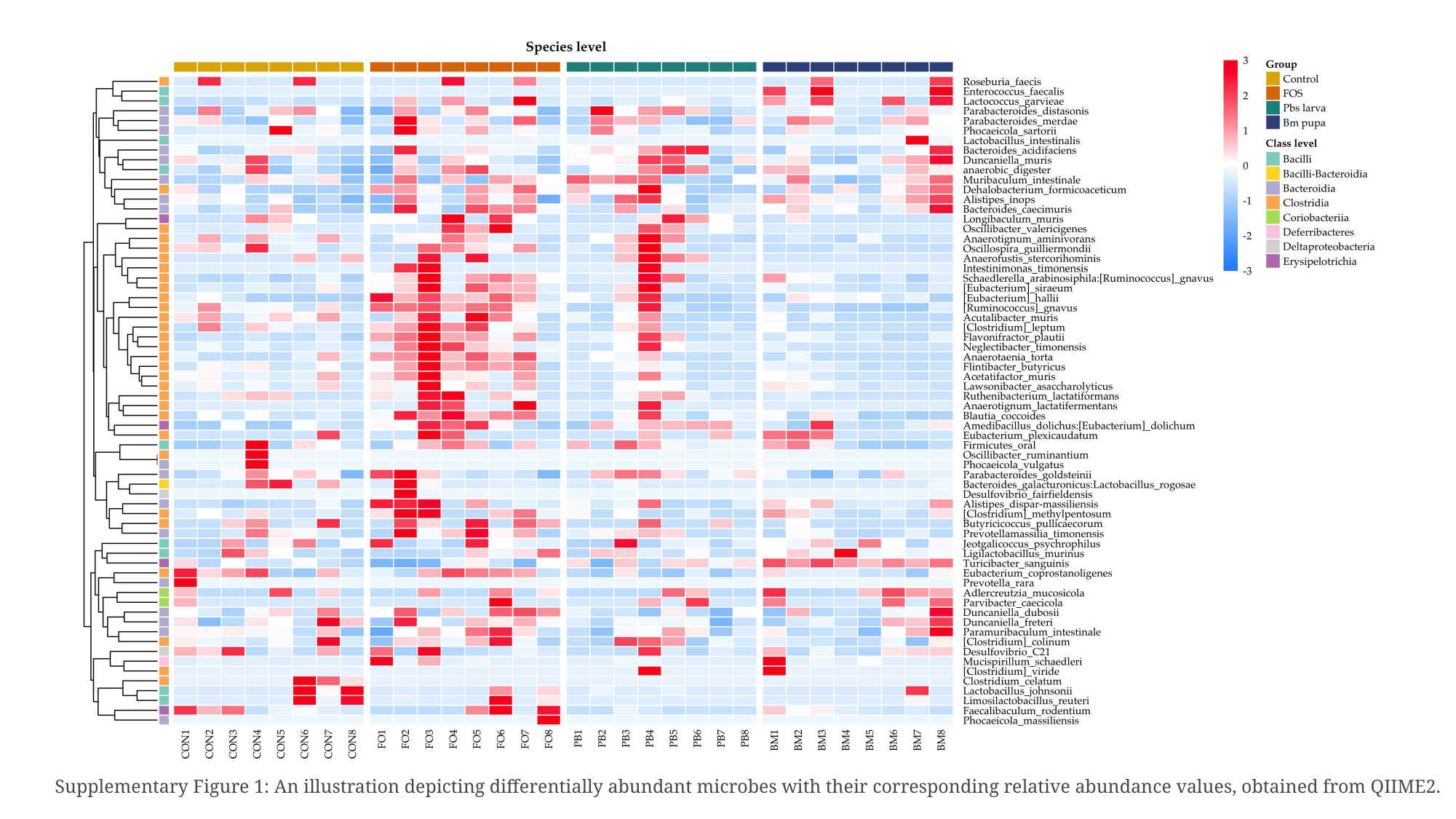

#
Supplementary Figure 1: An illustration depicting differentially abundant microbes with their corresponding relative abundance values, obtained from QIIME2.

## Slide 2
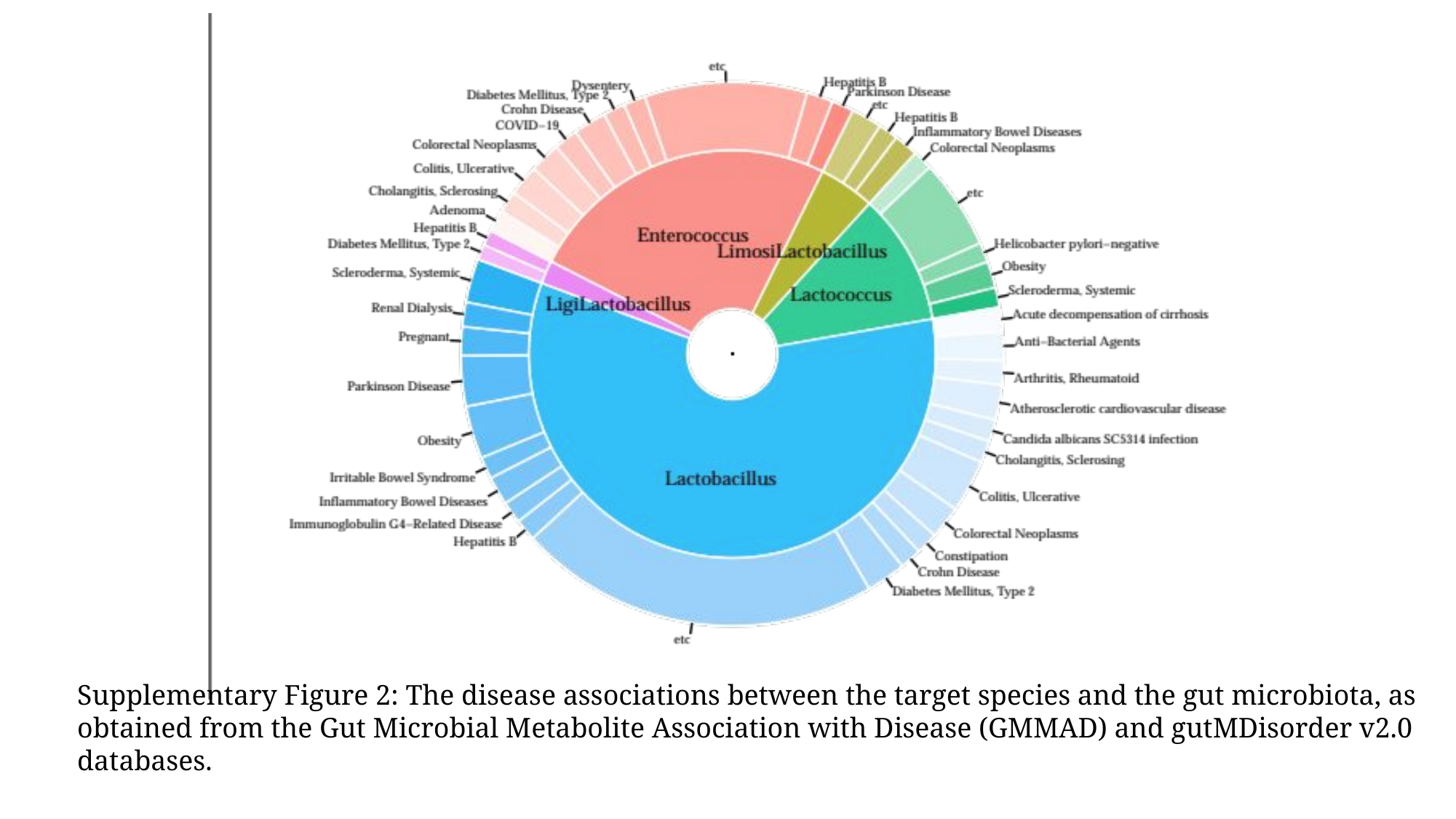

#
Supplementary Figure 2: The disease associations between the target species and the gut microbiota, as obtained from the Gut Microbial Metabolite Association with Disease (GMMAD) and gutMDisorder v2.0 databases.

## Slide 3
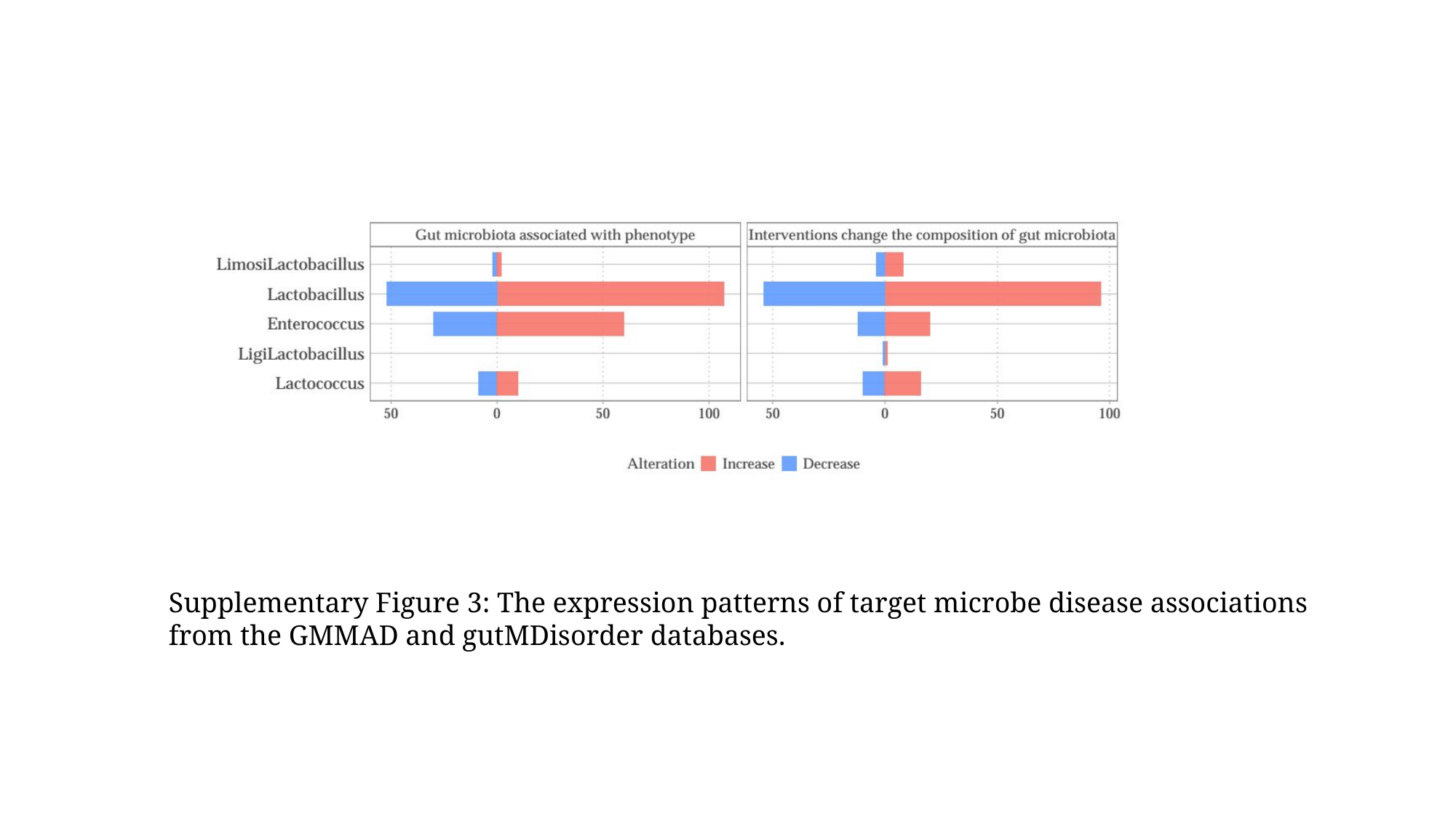

#
Supplementary Figure 3: The expression patterns of target microbe disease associations from the GMMAD and gutMDisorder databases.
